# Antifibrotic and molecular aspects of rifaximin in alcoholic liver disease: study protocol for a randomized controlled trial

**DOI:** 10.1186/s13063-018-2523-9

**Published:** 2018-02-26

**Authors:** Bjørn Stæhr Madsen, Jonel Trebicka, Maja Thiele, Mads Israelsen, Manimozhiyan Arumugan, Troels Havelund, Aleksander Krag

**Affiliations:** 1 Department of Gastroenterology and Hepatology, Odense University Hospital, Sdr. Boulevard 29, 5000 Odense C, Denmark; 2 OPEN, Odense Patient Data Exploratory Network, Odense University Hospital, J.B. Winsløws Vej 9 a, 3 Floor, 5000 Odense C, Denmark; 3 Department of Internal Medicine, University of Bonn, Sigmund Freud Str. 25, 53127 Bonn, Germany; 4 Institute of Clinical Research, University of Southern Denmark, Windsløwparken 19, 3 floor, 5000 Odense C, Denmark; 5 European Foundation for the Study of Chronic Liver Failure - EF Clif, Travessera de Gràcia, 11, 7th floor, 08021 Barcelona, Spain; 6 Institute for Bioengineering of Catalonia, C. Baldiri Reixac 10-12, 08028 Barcelona, Spain; 7 Novo Nordisk Foundation Center for Basic Metabolic Research, University of Copenhagen, Blegdamsvej 3B, 2200 Copenhagen, Denmark

**Keywords:** Liver fibrosis, Alcoholic liver disease, Gut microbiota, Rifaximin

## Abstract

**Background:**

Alcoholic liver disease is the leading cause of cirrhosis worldwide. Due to an increase in alcohol overuse, alcoholic liver disease has become an increased burden on health care systems. Abstinence from alcohol remains the cornerstone of alcoholic liver disease treatment; however, this approach is hampered by frequent relapse and lack of specific therapy for treating advanced cases of liver disease. In the present study, we hypothesized that gut microbiota drive the development of liver fibrosis and that modulation of gut microbiota with the gut-selective, nonabsorbable antibiotic rifaximin attenuates alcoholic liver fibrosis.

**Methods/design:**

Our double-blind, placebo-controlled trial will include 136 participants with biopsy-verified alcoholic fibrosis (Ishak liver fibrosis score of 1–4). Participants are randomized 1:1 to receive placebo or 550 mg of rifaximin twice daily for 18 months. A liver biopsy will be performed at the end of the treatment period to evaluate the effect of drug treatment on liver fibrosis. Stool, urine, and saliva specimens will be collected before treatment begins, at 1 month, and at the end of the treatment period. Fecal samples are used for microbiome deep sequencing. Changes in microbiome composition are compared before and after the trial medication period and linked to changes in liver fibrosis.

**Discussion:**

This is the first clinical trial to evaluate the effect of gut microbiota on liver fibrosis in humans. If gut microbiota are an important promoter of alcoholic liver disease, current results may open new therapeutic avenues and revolutionize the current understanding of chronic liver diseases.

**Trial registration:**

EudraCT, 2014–001856-51. Registered on 16 August 2014.

**Electronic supplementary material:**

The online version of this article (10.1186/s13063-018-2523-9) contains supplementary material, which is available to authorized users.

## Background

Alcohol consumption is the leading cause of liver cirrhosis in humans, and mortality from alcoholic liver disease compared to other common chronic diseases is on the rise [1, 2]. Unfortunately, research related to alcoholic liver disease is relatively underfunded and subject to limited interest from pharmaceutical companies; therefore, no new therapeutic interventions have been developed in the last few decades [3]. Furthermore, patients with this disease are often stigmatized, present a relatively high socioeconomic burden, and are a key societal challenge. Excessive alcohol consumption stimulates extracellular matrix production from hepatic stellate cells, which accumulates as liver fibrosis and replaces normal liver tissue. Although the liver is able to recover to some extent, sustained misuse of alcohol over several years can lead to progressive accumulation of liver fibrosis and eventually cirrhosis.

Due to its anatomical position, the liver is the first organ to encounter bacteria and bacterial products from the gut via portal venous blood. During alcoholic liver disease, intestinal microbiota change qualitatively and quantitatively in favor of a community with increased invasive potential, and the gut barrier is compromised [4]. As a consequence, an increased load of bacterial products are transported to the liver, where they activate Toll-like receptors, leading to proinflammatory gene expression and fibrogenesis [5]. This cross-talk between intestinal microbiota and the liver constitutes a gut-liver axis, which has been increasingly recognized as a key mechanism in the progression of alcoholic liver disease and pathogenesis of liver-related complications [6, 7].

### Study rationale

Modulation of gut microbiota using broad-spectrum antibiotics is currently being used in clinical hepatology to protect against complications associated with advanced liver diseases, such as bacterial translocation, spontaneous bacterial peritonitis, and recurrent episodes of hepatic encephalopathy [7]. Rifaximin, a nonabsorbable derivative of rifampicin, is currently being used to help treat traveler’s diarrhea and prevent recurrent episodes of hepatic encephalopathy in advanced liver disease [8]. Rifaximin exerts a broad-spectrum bactericidal effect against gastrointestinal bacteria by binding to the β-subunit of bacterial DNA-dependent RNA polymerase [9]. Previous studies have shown that rifaximin has a modest impact on the composition of gut microbiota. However, these studies were done in patients with end-stage liver disease using a 16S ribosomal RNA gene sequencing with limited microbiota sequencing depth [10, 11]. To date, there have been no studies evaluating the possible antifibrotic effect of modulating human gut microbiota with antibiotics. However, an increasing body of evidence from animal studies, including models of alcoholic liver disease, supports this notion [12, 13]. Due to its ability to selectively target gut microbiota with minimal systemic uptake [14] and its documented efficacy against complications related to advanced liver disease [8], as well as liver fibrosis in a preclinical model [13], we chose to use rifaximin to modulate gut microbiota in the present clinical trial.

### Study aim and hypothesis

We hypothesize that human gut microbiota and their metabolites are major promoters of fibrosis in alcoholic liver disease. Therefore, modulation of gut microbiota by rifaximin can attenuate or halt disease progression by decreasing the amount of profibrotic signals from gut microbiota reaching the liver.

## Methods/design

The present biopsy-controlled, double-blind study will include a total of 136 patients randomized 1:1 to receive a 550-mg tablet of rifaximin or placebo twice daily for 18 months. Randomization is being performed in blocks of four, with stratification according to the Metavir fibrosis score from initial liver biopsies and whether or not participants abstained from alcohol for 6 months prior to study onset. Participants are each assigned a consecutively numbered three-digit identification number in the trial. Each identification number is by chance allocated to either placebo or rifaximin. The randomization list is generated electronically by a central computer stored at the pharmacy at Odense University Hospital (Odense, Denmark); only authorized pharmacy personnel know the randomization code. Sponsors, investigators, nurses, laboratory assistants, and/or personnel involved in trial participant care have no knowledge of the randomization. Sealed, opaque envelopes with randomization keys for each participant are being kept at Odense University Hospital. Sponsors and investigators have access to the coded envelopes at all times in case unblinding is necessary. Placebo tablets will be similar in size, shape, and weight to the rifaximin tablet to secure concealment. Pre- and post-treatment biopsies are assessed together in a blinded manner by one expert pathologist. Fecal samples are collected at home and frozen immediately in a home freezer. They are transported to the clinic in ice where they are stored at –80 °C. Samples are processed for shotgun metagenomics sequencing of the gut microbiota using the recommended procedures by the International Human Microbiome Standards Consortium [15]. Saliva samples are collected at the clinic in two tubes. One tube is frozen to –80 °C immediately. The other is stored at +5 °C for 3 h before freezing to –80 °C. Microbial DNA extraction is performed with a Macherey-Nagel NucleoSpin Kit, and 16S ribosomal RNA gene sequencing of the oral microbiota is performed by amplifying the V4 hypervariable region using standard procedures [16]. Urine samples are collected to detect the presence or absence of ethyl glucuronide, a marker of alcohol consumption. For further information, see the populated Standard Protocol Items: Recommendations for Interventional Trials (SPIRIT) checklist in Additional file 1.

### Primary outcome measure

The primary outcome measure is the proportion of patients with an improvement in their liver biopsy Ishak (fibrosis) score greater than or equal to 1.

### Secondary outcome measures

The secondary outcome measures are as follows:

- Markers of fibrosis: collagen proportionate area, enhanced liver fibrosis score, Pro-C3, and hydroxyproline level
- Markers of matrix remodeling: tissue inhibitor of metalloproteinase 1, matrix metallopeptidase 2, and profibrotic cytokines (transforming growth factor β1, platelet-derived growth factor β, and connective tissue growth factor)
- Proinflammatory cytokines: tumor necrosis factor α, monocyte chemoattractant protein 1, and cluster of differentiation 163
- Liver stiffness assessed by transient and shear wave elastography
- Changes in the composition of the gut microbiota assessed by shotgun metagenomics sequencing
- Quality of life assessed by the Short Form (36) Health Survey and Chronic Liver Disease Questionnaire
- Nutritional status assessed by weight and hand grip strength

### Recruitment and enrollment of participants

Participants are being recruited from an ongoing observational study (*Liver Disease in Alcohol Overusing Patients*) at the department of Gastroenterology and Hepatology, in which liver biopsies were taken from 400 patients with alcohol overuse (defined as the use of ≥ 24 g alcohol/day for women and ≥ 36 g alcohol/day for men; approved by regional committees on health research ethics for southern Denmark, project ID S-20120071). Among these, a subset of 136 patients will be enrolled in the current study if they comply with the inclusion and exclusion criteria outlined below.

#### Inclusion criteria

The inclusion criteria are as follows:

1. Women of child-bearing age and potential should use safe contraception and provide a negative pregnancy test during the study period.
2. Patients with a liver fibrosis Ishak score of 1–4.

#### Exclusion criteria

The exclusion criteria are as follows:

1. Patients with a known allergy to rifaximin
2. Patients whom the investigator judges will not be or are not compliant with the trial treatment protocol
3. Patients who received antibiotic treatment of any kind 4 weeks prior to study onset

#### Study withdrawal

Patients will be withdrawn from the study for the following reasons:

1. If blinding is repealed
2. If the patient undergoes treatment with another antibiotic for more than 4 consecutive weeks or four times during the study period
3. If the trial participant withdraws written consent
4. If the investigator judges that withdrawal is in the participant’s best interest

#### Study dropouts

Trial participants will be classified as dropouts if they meet either of the following criteria:

1. The trial participant ingests less than 75% of the planned treatment.
2. The trial participant does not attend planned visits, despite contact by telephone, letter, or mail.

### Sample size calculation

According to a previous trial, alcoholic liver fibrosis was calculated to regress in 27% of patients, remain unchanged in 57%, and progress in 16% over a 2-year period when patients with hepatitis C were excluded [17]. Therefore, we estimated the natural history of regression to be approximately 14% during an 18-month period for our study population. The smallest relevant difference is considered to be a 25% absolute increase in the percentage of patients who regress by 1 point or more in their histological score. If the risk of performing type 1 and type 2 errors is set to 5% and 20%, respectively, then 136 patients are needed when one performs a power calculation using a two-sided test; this includes a dropout rate of 20%. Both intent-to-treat and per protocol analyses will be performed.

### Measurements and investigations

**Fig. 1:**
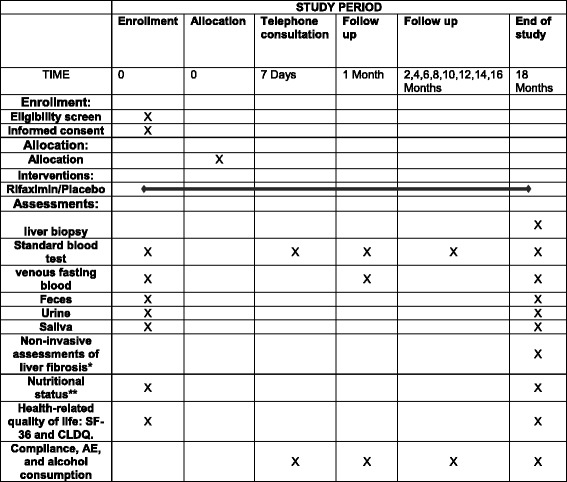
Measurements and investigations. Legend section: *SF-36* Short Form (36) Health Survey, *CLDQ* Chronic Liver Disease Questionnaire, *AE* adverse event, *by transient and shear wave elastography, **by weight and hand grip strength

Measurements and investigations follow the study plan outlined in Fig. 1.

During follow-up, the investigators or a study nurse will keep a record of the medication and hand out new trial medication to participants in the outpatient clinic (Fig. 1); both used and unused trial medication is registered to keep track of the amount ingested by each participant. In cases of uncertainty regarding the amount of medicine ingested, the highest dose definitively known will be registered. During the planned follow-up, adverse events, alcohol consumption, and use of antibiotics will be routinely registered in the case report form.

### Drug information

Rifaximin is a semisynthetic analog of the antimicrobial rifampicin that has an excellent safety profile and selective impact on gut microbiota [10, 11, 18]. The tolerability of long-term rifaximin use in patients with liver disease has proven to be good in a previous study involving 299 patients. In that study, participants were randomized to placebo (*n* = 159) or 550 mg of rifaximin (*n* = 140) twice a day for 6 months in order to prevent recurrent hepatic encephalopathy in patients with cirrhosis [8]. Their results showed that rifaximin significantly reduced the risk of a breakthrough episode of hepatic encephalopathy from 46% (placebo) to 22% (rifaximin). The majority of reported side effects were of gastrointestinal origin, including nausea and abdominal discomfort. The incidence of moderate and serious adverse events was similar in patients receiving placebo or rifaximin [8].

### Risks and disadvantages for participants

Percutaneous liver biopsy is considered a safe investigation with few complications when used to diagnose nonmalignant hepatic disorders. However, most patients experience some pain related to the puncture site. Potential risks related to the procedure are intra-abdominal bleeding, bile peritonitis, and perforation of the gallbladder. The mortality rate is 1 in 10,000; however, no deaths have been directly related to the procedure when it is used to help diagnose the cause of an abnormal liver function test. Major bleeding episodes (the most frequent complication) appear in 2.2 out of 1000 biopsies in patients who are under further examination due to an abnormal liver function test [19]. To minimize risk to the current trial participants, this procedure will only be done by trial investigators or medical staff with extensive experience. Furthermore, there is no discomfort or risk related to B-mode abdominal ultrasonography or ultrasound elastography.

### Ethics

Currently there are no known effective drugs to treat fibrotic liver disease, and the effects of rifaximin remain unclear. Hence, no patient will be withheld medication with proved efficacy by participating in this trial. All patients should continue their regularly prescribed medications during the trial. Patients receiving placebo are unlikely to experience any improvements in their disease or symptoms due to the treatment *per se*. However, it is necessary to treat half of the patients with placebo in order to ascribe observed effects to rifaximin with certainty. All patients are at risk for disease progression and will benefit from close follow-up and early detection of advanced liver disease by undergoing a liver biopsy. During the trial, participants will be encouraged to reduce their alcohol consumption. As suggested by a previous study, many patients who overuse alcohol included in clinical trials on alcoholic liver disease stop drinking or reduce their alcohol consumption, causing spontaneous improvement of their liver disease symptoms [20]. Overall, we believe the benefits of participating in this trial outnumber the risks, and we also believe it to be ethically safe. Informed consent is obtained from all participants.

## Discussion

The present clinical trial is an investigator-initiated, randomized, double-blind, placebo-controlled investigation into the effect of rifaximin on human liver fibrosis. This proof-of-concept trial is aimed at elucidating the role of profibrotic cross-talk between gut bacteria and the liver. If gut bacteria are significant contributors to liver fibrosis, modulation of gut flora could be a novel way to improve outcomes in liver disease.

### Trial status

The trial started enrolling participants March 2015. By August 2017, 66 participants were enrolled and 25 had completed the study. The last patient (*n* = 136 patients) will be enrolled in December 2018. The estimated end of follow-up and trial shutdown is June 2020.

## Additional file

Additional file 1: SPIRIT 2013 checklist: recommended items to address in a clinical trial protocol and related documents. (DOC 121 kb)
